# Efficient Estimation of the Robustness Region of Biological Models with Oscillatory Behavior

**DOI:** 10.1371/journal.pone.0009865

**Published:** 2010-04-01

**Authors:** Mochamad Apri, Jaap Molenaar, Maarten de Gee, George van Voorn

**Affiliations:** 1 Biometris, Department of Mathematical and Statistical Methods, Wageningen University, Wageningen, The Netherlands; 2 Netherlands Consortium for Systems Biology, Amsterdam, The Netherlands; Fondazione Telethon, Italy

## Abstract

Robustness is an essential feature of biological systems, and any mathematical model that describes such a system should reflect this feature. Especially, persistence of oscillatory behavior is an important issue. A benchmark model for this phenomenon is the Laub-Loomis model, a nonlinear model for cAMP oscillations in Dictyostelium discoideum. This model captures the most important features of biomolecular networks oscillating at constant frequencies. Nevertheless, the robustness of its oscillatory behavior is not yet fully understood. Given a system that exhibits oscillating behavior for some set of parameters, the central question of robustness is how far the parameters may be changed, such that the qualitative behavior does not change. The determination of such a “robustness region” in parameter space is an intricate task. If the number of parameters is high, it may be also time consuming. In the literature, several methods are proposed that partially tackle this problem. For example, some methods only detect particular bifurcations, or only find a relatively small box-shaped estimate for an irregularly shaped robustness region. Here, we present an approach that is much more general, and is especially designed to be efficient for systems with a large number of parameters. As an illustration, we apply the method first to a well understood low-dimensional system, the Rosenzweig-MacArthur model. This is a predator-prey model featuring satiation of the predator. It has only two parameters and its bifurcation diagram is available in the literature. We find a good agreement with the existing knowledge about this model. When we apply the new method to the high dimensional Laub-Loomis model, we obtain a much larger robustness region than reported earlier in the literature. This clearly demonstrates the power of our method. From the results, we conclude that the biological system underlying is much more robust than was realized until now.

## Introduction

It is remarkable but well-known that many biological systems are robust under vastly different conditions [1], [2]. Although these systems might experience strong internal or external perturbations, e.g., through environmental changes or noise, they still operate reliably. This is, for example, observed in chemotactic behavior and patterning development [2]. Robustness is an essential feature of biological systems [3], [4], and any mathematical model describing their behavior should also have this property [5]. This implies the need for an efficient tool to analyze the robustness of these models.

Here we focus on the parametric robustness of biological models that show oscillatory behavior. Oscillations are ubiquitous in biology. It is found, for example, in the pulse of the heart, the circadian rythm, and the signal transduction that involves adenosine 3′,5′-cyclic monophospate (cAMP) in the chemotactic of *Dictyostelium discoideum* [6]. The robustness of a model is determined by answering the question how far the parameters of the model could be perturbed so that the qualitative behavior of the system does not change. An example of such a change is, e.g., the transition from oscillatory behavior to a steady state equilibrium. Such a drastic transition is called a *Hopf bifurcation*. There are many types of bifurcations possible in dynamical biological models.

Given a so-called *nominal point* in parameter space for which a system has a stable periodic solution, in general a region around this point exists within which the system oscillates. We call such a region a “robustness region” if no bifurcation of any kind occurs in its interior and if in each point of its boundary the system undergoes some bifurcation. The type of the latter bifurcations may be of any kind. An important consequence of this definition is that the period of the oscillations varies smoothly over the robustness region. If somewhere a period doubling bifurcation (also referred to as flip bifurcation) occurs, such a dramatic change in qualitative behavior indicates that the system is no longer robust. According to our definition we meet in such a point the boundary of the robustness region. (Note that in this paper, the words flip bifurcation and period doubling bifurcation are used interchangeably.)

In the literature, some methods have been proposed to analyze robustness of models with oscillatory behavior. Robustness with respect to perturbations of a single or at most two parameters simultaneously can be investigated using a bifurcation analysis package such as AUTO [7]. With this package, the boundary of the robustness region can be obtained. In most cases, however, more parameters are involved and AUTO is no longer applicable. In [8], the Structured Singular Value method (SSV) from control theory [9] was used to quantify the robustness of the Laub-Loomis model [6]. This model has an oscillatory solution for a specific set of parameter values, the so-called nominal values. It was investigated how much the nominal values might be changed before a bifurcation would occur. The authors initially claimed that the allowed maximum parameter variation is 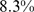. The work was then improved by applying a hybrid optimization method which yielded a much smaller variation of 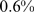 [10]. Ghaemi et al. utilized a Routh-Hurwitz stability criterion that resulted in 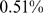 variation for the Laub-Loomis model [11]. The percentage values of parameter variations that are presented in these papers suggest that all parameters have the same sensitivity. However, the model might be more sensitive to some parameters than to others [12]. Furthermore, the authors studied only the Hopf bifurcation that occurs when the stable periodic behavior turns into an equilibrium.

Here we present an alternative method to analyze the parametric robustness of biological models with stable oscillatory behavior (also referred to as “periodic solution” or “limit cycle”). The method aims at finding an approximation for the whole robustness region, taking into account that the sensitivity of the system might be highly parameter dependent. The consequence is that in our approach it is not useful to report the resulting estimate in terms of a percentage of the nominal value. On the contrary, the robustness region often turns out to be far from symmetric around the nominal point. Furthermore, the present approach allows for the detection of any kind of bifurcations, and is not limited to Hopf bifurcations. Another aspect refers to dimensionality. In high-dimensional systems, an important feature of any numerical method is efficiency. Many methods suffer from the so-called “dimensional curse”, i.e. the computing time scales exponentially with dimension. For example, if we would use a Monte-Carlo approach for estimating the shape of robustness regions, we would certainly be confronted with this limiting factor. However, the method presented here has the computational advantage that it scales linearly with the number of parameters. That is the reason that it is highly efficient for systems with a high-dimensional parameter space.

The present method is based on Floquet theory and continuation of the periodic solution. Starting from the nominal parameter set, we construct an estimate for the robustness region by scanning the parameter space in orthogonal directions. If necessary, the obtained estimate is refined by shifting the nominal point to a carefully chosen new position. We do not only focus on Hopf bifurcations, but take into account all types of bifurcation that might occur to periodic solutions, including period doubling and Neimark-Sacker bifurcations. So, also bifurcations that lead to chaotic behavior may be detected. In addition, the presented method yields extra information such as the period and the amplitude of the solution for free.

To demonstrate the ideas and power of the proposed method, we apply it to ecological and biological network models that admit stable periodic solutions: the Rosenzweig-MacArthur (RMA) model and the Laub-Loomis (LL) model. The RMA model is chosen for illustrational purposes. It is well known for its rich bifurcation pattern and serves as a test case here. It is a low dimensional system for which our method is not especially designed, but it serves as a useful check of performance. It consists of three state variables with six parameters where two of them are taken free. The LL model is a high dimensional system that consists of seven state variables with fourteen parameters. Its robustness has been already investigated in [8], [10], [11]. As an extra check on low dimensional systems we analyze the LL model with twelve fixed and only two parameters perturbed. Our results for two dimensional systems fully agree with those obtained with existing approaches. The results for the high dimensional LL model clearly demonstrate that the present method is a real extension of the existing approaches.

## Results

The stability of a periodic solution can be verified using Floquet theory (see [13] and [14]). In this theory, the Floquet multipliers, which are the eigenvalues of the so-called *monodromy*-matrix, are used to indicate stability. One of the Floquet multipliers is always real and equal to 1. A necessary and sufficient condition for a periodic solution to be stable is that the modulus of the other Floquet multipliers is less than 1, i.e., they lie inside the unit circle in the complex plane. If the parameters are perturbed and one of the multipliers crosses the unit circle, the solution looses its stability and a bifurcation happens. This bifurcation can be of several types as discussed in the Material and Method section.

This suggests that in order to analyze the robustness of oscillatory behavior of a model, we only need to observe its Floquet multipliers as functions of the parameters. In the Material and Method section, we describe the details to find in an efficient way an estimate for the robustness region. Starting in a so-called nominal point in parameter space for which a stable periodic solution exists, the parameter space is scanned along orthogonal directions to detect where along these lines bifurcations occur. This yields an initial estimate of the robustness region, that is gradually improved by shifting the nominal point and varying the directions.

In the next sections, we apply our method to two biological models: the low-dimensional RMA model and the high-dimensional LL model.

### Application to the Rosenzweig-MacArthur Model

The Rosenzweig-MacArthur (RMA) model is an ecological model that describes the time evolution of a predator-prey system [15]. In dimensionless form, this 3-dimensional model reads as
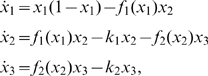
(1)where
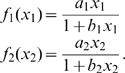
(2)Here, 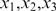 denote the prey, predator, and top predator populations, respectively, 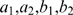 are the parameters in the Michaelis-Menten functions 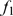 and 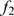, and 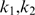 are death rate parameters.

**Figure 1:**
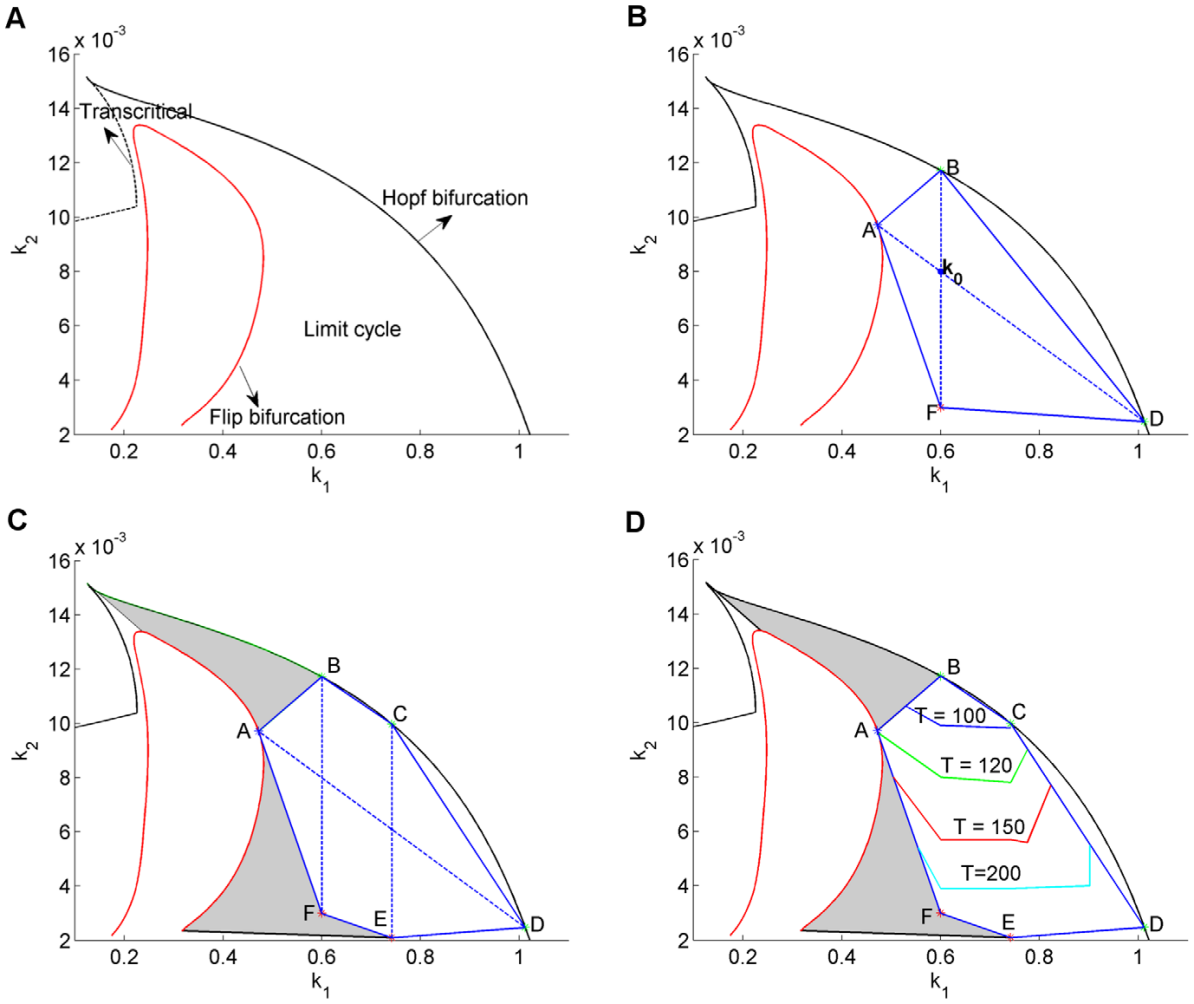
Bifurcation diagram and successive approximations of the robustness region of the RMA model. (A) Bifurcation diagram as a function of the death rate parameters 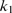 and 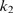 [17]. (B) Initial approximation. (C) Second approximation. (D) Estimated level lines of the period of the periodic solution. Note that the scale for 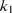 and 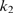 is not the same, which is the reason the orthogonality of the lines AD and BF is not directly clear from the picture. The shaded areas in (C) and (D) indicate regions where an infinitely number of period doubling bifurcations are located.

The dynamical behavior of this model for the fixed coefficient values
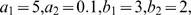
(3)has been extensively investigated in [16]–[18] as a function of 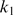 and 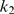. The resulting bifurcation diagram is depicted in Figure 1A. From this figure, we see that the limit cycle behavior of the model may experience a Hopf bifurcation, a transcritical bifurcation, or may transform into a flip bifurcation. Since there are infinitely many flip bifurcations in this bifurcation diagram, it is not possible to indicate all their positions in Figure 1A. Therefore, as a warning, we shade some areas in Figure 1C to indicate that flip bifurcations may occur somewhere in these areas. Due to infinitely many flip bifurcations, we restrict ourselves to the positions of the first period doubling bifurcations, which lie on the red curved line.

**Figure 2:**
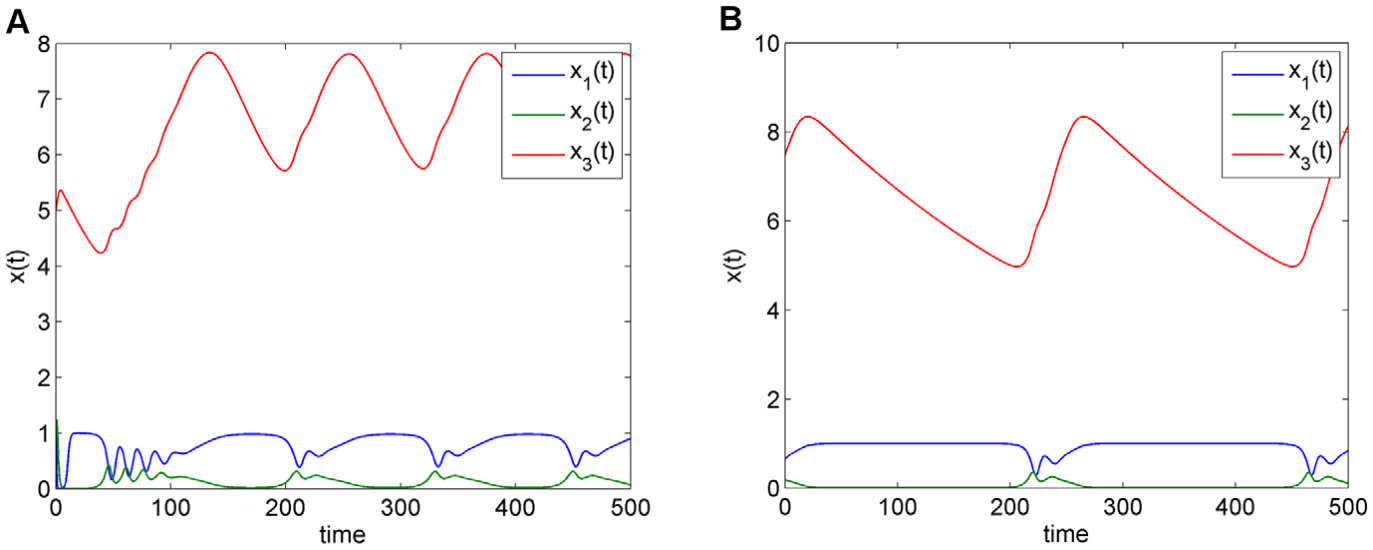
Behavior of the limit cycle solution of the RMA model. (A) In nominal point 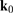. (B) In point F.

We apply our method to show how an estimate is obtained for the region in Figure 1A where a stable limit cycle exists. As nominal starting point we take 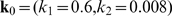. In 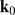, the solution converges to a periodic solution with period 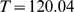 as shown in Figure 2A. The corresponding Floquet multipliers are
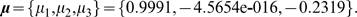



We notice that the largest multiplier 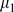 is indeed equal to 1 within the numerical accuracy. 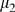 and 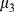 lie inside the unit circle, so the limit cycle in 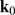 is stable. Following the method described underneath and summarized in equations (23)–(27), we construct two orthogonal directions, 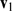 and 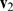, and perturb the nominal parameter set 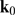 in these directions. The direction 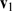 is chosen such that the Floquet multipliers will change mostly when moving along 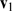 in the 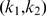 plane and 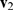 is orthogonal to 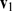.

Continuation is applied along perturbation direction 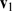 until points B, denoted by a green star, and F, denoted by a red star, in Figure 1B are reached. Continuation is stopped at point B because the multipliers at that point are
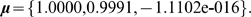
So, 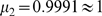 and this indicates that the method has successfully found a fold bifurcation. Using only Floquet multipliers, one cannot discriminate between a tangent, for which the cycle collides with a saddle limit cycle, and a Hopf bifurcation, for which the limit cycle disappears into an equilibrium. However, since in both cases the boundary of the robustness region is reached, this is not a problem at all. Just for curiosity we used AUTO to confirm that it is the latter option. Continuation is stopped at point F. It does not make sense to continue beyond this point, since the value of parameter 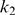 is so small there, that it is already hardly acceptable from a biological point of view. This also manifests itself in a very long period and a highly irregular shape of the limit cycle, that gives rise to a very long computational time. An example is given in Figure 2B, where we show the time behavior of the components at point F.

When the continuation procedure is applied along direction 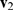, the method hits two bifurcation points, A and D. At point A, the mutlipliers are
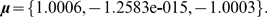
We notice that 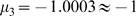, and we conclude that the method has successfully found a flip bifurcation, which is denoted by a blue-star. Since a flip indicates a possible route to chaos and it means the end of the limit cycle, as meant in the definition of robustness, this is also a boundary of robustness. On the other hand, we detect point D as a Hopf bifurcation. Thus, we obtain region ABDF as our first, crude approximation of the robustness region of the model. Note that the orthogonality of 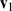 and 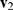 that leads to the axes AD and BF is not directly clear from Figure 1B, because the axes have different scales.

Next, an improvement on this initial approximation is obtained by shifting the nominal point 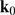 to the midpoint of the longest axis, in this case the midpoint of AD which is denoted by 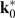 in Figure 1C. Applying the continuation procedure to the shifted nominal point 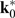 along the direction 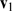, we obtain a new axis CE. Here, point C is a Hopf bifurcation point. Just as for point F, we stop continuation in E since the value of 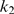 becomes too small. Together with the previous findings, we now obtain the bigger estimating region ABCDEF, as shown in Figure 1C.

During the calculations, we simultaneously obtain a lot of information on the period and the shape of the limit cycle. In fact, this information is available along all the lines through 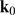 and 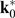. In Figure 1D, this info is used to draw level lines for the period. It provides a nice indication how the period behaves as a function of the parameters. Since the RMA model only serves as a low-dimensional illustration of the ideas behind the proposal estimation algorithm, we will not refine the approximation further, but rather turn to a high-dimensional example.

### Application to the Laub-Loomis Model

**Figure 3:**
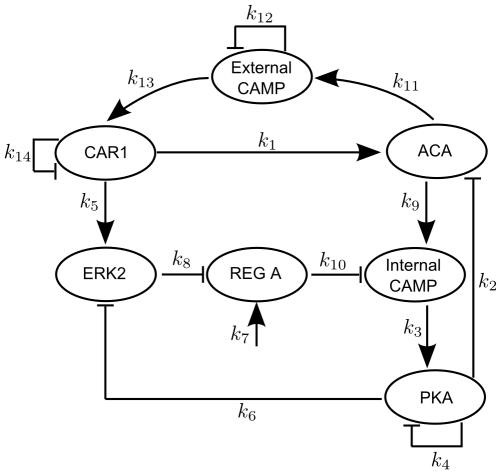
The network underlying the Laub-Loomis model.

The Laub-Loomis (LL) model [6] describes the dynamical behavior of the molecular network underlying cAMP (adenosine 3′,5′-cyclic monophospate) oscillation observed in population of *Dyctiostelium discoideum* cells. The molecular network is depicted in Figure 3.

Here, after the binding of extracellular cAMP to the surface receptor CAR1, adenylate cyclase (ACA) activates internal cAMP. When internal cAMP is accumulated, it activates protein kinase PKA. In addition, ligand-bound CAR1 also activates the MAP kinase ERK2, which is then inactivated by PKA. Therefore, ERK2 no longer inhibits the cAMP phosphodiesterase REG A. A protein phosphatase activates REG A such that REG A can hydrolyze internal cAMP, hence the concentration of internal cAMP is reduced. When the internal cAMP is hydrolyzed by REG A, PKA activity is inhibited by its regulatory subunit, so that both ACA and ERK2 activities go up.

Based on the network above, the spontaneous oscillation in cAMP is a solution of the following model
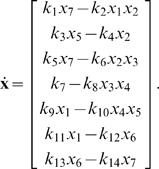
(4)Here, the state variable 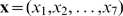 represents the concentrations of seven proteins: 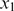 = [ACA], 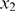 = [PKA], 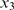 = [ERK2], 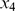 = [REG A], 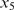 = [Internal cAMP], 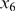 = [External cAMP], and 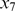 = [CAR1]. The model has 14 parameters, incorporated in the parameter vector 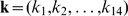.

**Figure 4:**
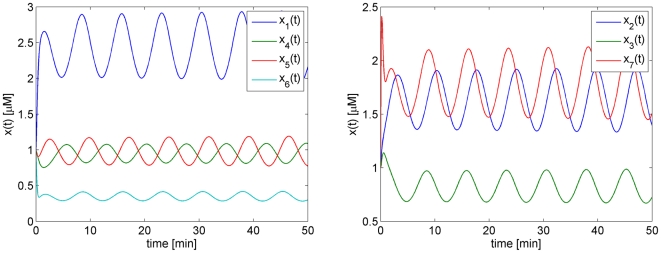
Periodic solution of the Laub-Loomis model (4) at the nominal parameter values in Table 1.

At the nominal parameter set in Table 1, which is denoted by 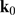, this system has a stable periodic solution. Thus, if we choose the initial concentrations within the basin of attraction, the solution will converge to this periodic solution, as shown in Figure 4.

**Table 1:** Nominal values for 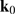 the Laub-Loomis model (from [8], [10], [11]).

Parameter	Units	Nominal value
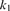	min 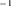	2.0
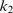	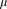 M 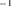 .min 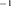	0.9
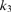	min 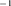	2.5
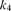	min 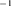	1.5
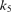	min 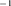	0.6
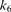	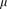 M 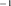 .min 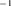	0.8
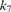	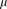 M.min 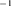	1.0
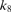	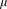 M 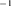 .min 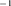	1.3
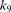	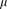 M 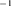 .min 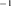	0.3
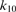	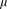 M 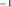 .min 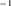	0.8
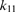	min 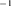	0.7
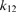	min 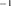	4.9
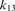	min 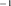	23
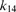	min 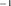	4.5

We found that the periodic solution at the nominal parameters 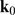 has period 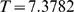 and the multipliers are given by
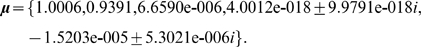



We notice that the largest multiplier, 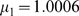, is equal to 1 within the numerical accuracy. Since the second largest multiplier 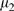 is also quite close to 1, we expect that the nominal point 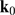 is close to a bifurcation point.

#### Restriction to a 2-dimensional cross-section of parameter space

**Figure 5:**
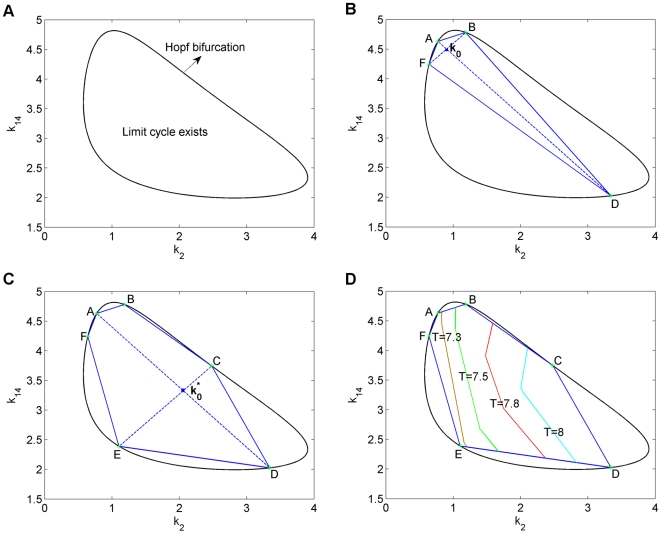
Cross-section of the robustness region of the Laub-Loomis model in the 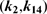 plane. (A) Result by AUTO. (B) First approximation based on 4 boundary points. (C) Second approximation based on 6 boundary points. (D) Level lines of the period of the periodic solution.

**Figure 6:**
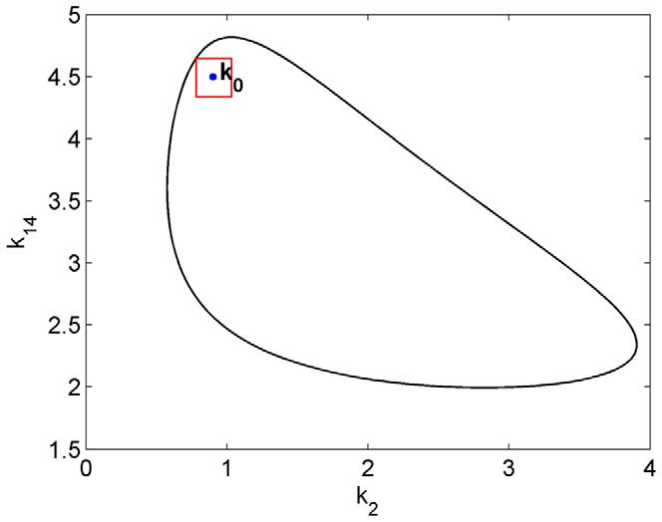
Robustness region of the Laub-Loomis model in parameter space. Black-line from AUTO, the red box indicates the estimate that would be obtained if the methods published earlier and developed for high-dimensional system [8], [10], [11] would be applied to the two dimensional case, in which only 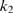 and 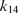 are varied.

For illustrational purposes, we first fix 12 parameters setting them at the values in Table 1 and only vary 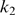 and 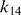. In this way we deal with a two dimensional cross-section in the high-dimensional parameter space. The advantage is that the results can be compared to results obtained with AUTO and in [11]. AUTO yields the robustness region given in Figure 5A. This region perfectly agrees with the region reported in [11]. However, it should be noted that the method in [11] yields a very good estimate only in the two-dimensional case. For higher dimensions, their approach leads to a much more restricted estimated region. If we would apply the more-than-two-dimensions approach in [11] or in [8], [10] to the present two-dimensional case, we would only find the small square shaped estimate indicated in Figure 6.

Applying the algorithm in (23)–(27), we obtain two directions: 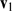, which is the most sensitive direction; and 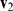, which is orthogonal to 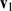. Along these directions, we perform the continuation procedure. This leads to our first approximation of the robustness region ABDF as shown in Figure 5B.

As denoted in the figure, our method successfully detected the four fold bifurcation points, A, B, D, and F which are indicated with green stars. According to the results obtained by AUTO, these points are Hopf bifurcations points where the second largest modulus of multipliers is very close to 1. For instance, at point A
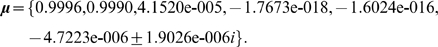
We notice that the initial approximation is much smaller than the real robustness region found by AUTO. We improve our approximation by shifting the nominal parameter 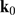 to 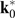, the midpoint of AD. When the continuation procedure is applied to the new nominal parameter 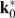 along direction 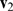, we find the Hopf bifurcation points C and E. Together with the first approximation, we now have obtained the larger approximation region ABCDEF, as shown in Figure 5C. As extra information, we get for free the level lines for the period as indicated in Figure 5D. The approximation could be further improved by taking more perturbation directions, but this is hardly necessary to get a very good impression of the robustness region.

#### Application in full-dimensional parameter space

Let us now investigate the robustness region of the Laub-Loomis model in the 14-dimensional parameter space. It will be clear that in this high-dimensional case the results are hard to present visually. According to algorithm (23)–(27), we find 14 orthogonal directions 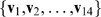 which, for convenience, are normalized to have unit length.

**Figure 7:**
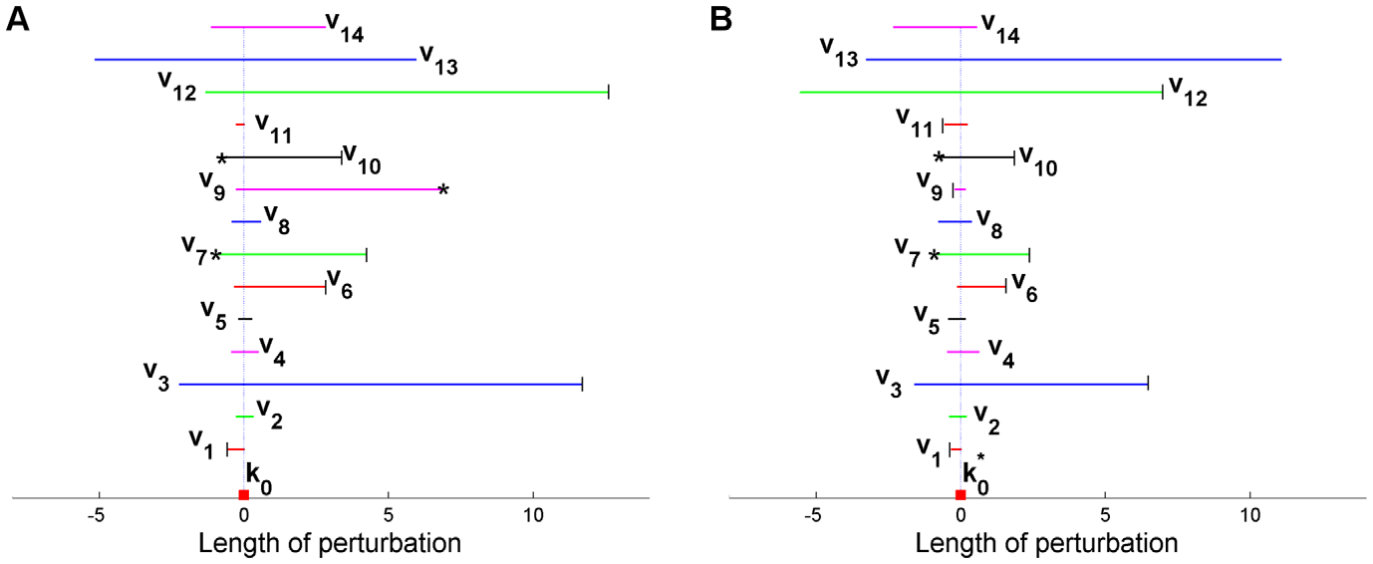
Representation of the “width” of the robustness region of the LL model. This region is measured along the 14 orthogonal directions 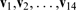 used to scan the parameter space. In (A), these directions start in nominal point 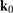 (see Table 1). In (B), the directions start in 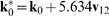. If an end point is marked with 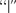, one of the parameters has become close to zero. If an end point marked with “*”, the period of the limit cycle becomes extremely long. If an end point does not have mark, a fold bifurcation is detected. The lengths of the horizontal lines indicate how far this direction can be followed in negative and positive directions so that a stable limit cycle is found. All directions are normalized to have unit length. A step of, e.g., length 6 in 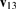 direction in (A) means that the unit vector in this direction can be made 6 times longer before a bifurcation is detected.

**Figure 8:**
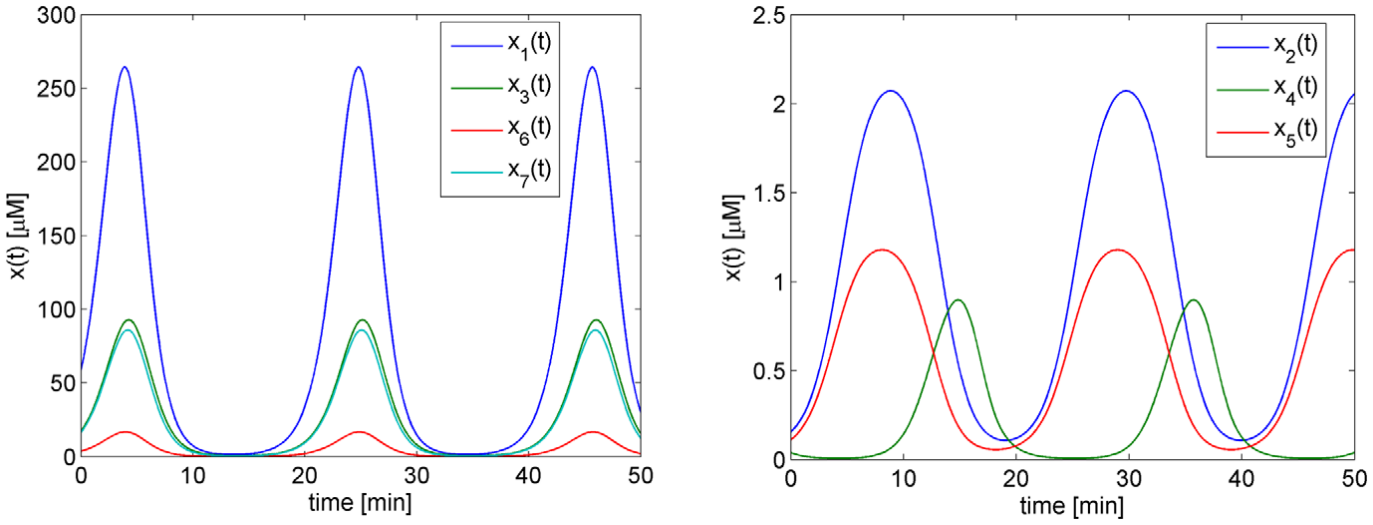
Limit cycle behavior of the Laub-Loomis model for parameter vector 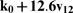. These parameter values are given in Table 2.

By applying continuation and observing the multipliers during the continuation, we easily obtain an estimate of the 14-dimensional robustness region. This estimate is shown in Figure 7A in a dedicated form. In this figure, the range of perturbations that is allowed to maintain the stability of the limit cycle is shown by horizontal lines for each perturbation direction. There are three possibilities that we stop the continuation: one of the perturbed parameters becomes close to 0 (in the LL model, all parameters should be positive), a bifurcation is detected, or the limit cycle gets an extremely long period. In the latter case, we need too much computational time to approximate the limit cycle. If one of the parameters becomes close to 0, we denote in Figure 7 the point by 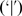; if a bifurcation is detected, we do not put any marker on the point; and if the continuation is stopped because of computing time, we denote the point by (*). For example, in the 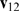 direction the nominal parameter 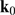 can be perturbed in the range
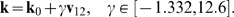
(5)The continuation is stopped at 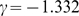 because then a fold bifurcation is detected, which follows from the Floquet multipliers
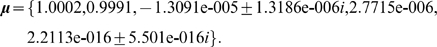
At 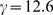, the system still admits a stable limit cycle behavior as shown in Figure 8, but we stop the continuation because one of the perturbed parameters becomes very close to 0, see Table 2.

**Table 2:** Perturbed parameter 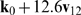.

Parameter	Units	Perturbed value
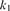	min 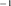	2.6982
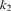	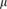 M 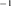 .min 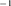	0.9330
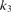	min 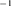	2.4641
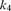	min 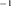	1.3871
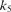	min 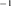	0.7495
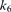	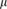 M 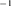 .min 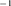	0.6507
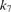	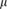 M.min 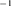	0.9006
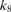	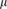 M 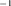 .min 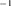	1.3690
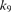	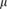 M 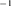 .min 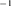	0.0009
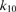	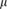 M 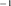 .min 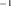	0.6758
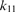	min 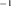	1.1100
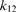	min 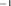	17.4668
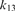	min 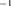	23.0125
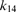	min 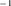	4.4666

**Figure 9:**
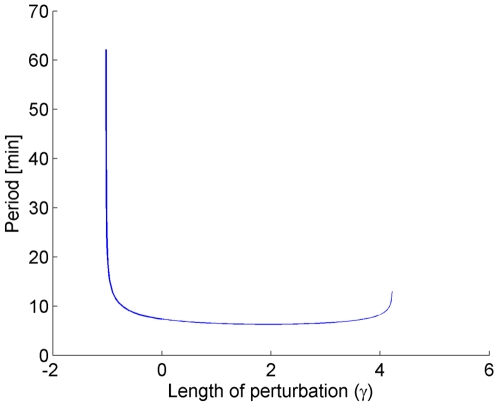
Behavior of the limit cycle period in the LL model along the 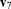 direction. Note that the period dramatically increases in the vicinity of 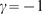.

In the 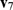 direction, the nominal parameter can be perturbed in the range
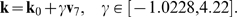
(6)Continuation is stopped at 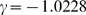 because the period of the limit cycle becomes extremely long and requires too much computational time. The behavior of the period along this direction is shown in Figure 9. At 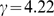, the continuation is stopped because one of the perturbed parameters becomes very close to 0.

To get still a better impression of the robustness region, we shift the nominal parameter. From the result in Figure 7A, we find that the system can be mostly perturbed in the direction of 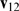. Therefore, we shift the nominal point 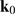 to the midpoint of this axis, and we denote the new nominal point by 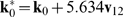. When the method is applied to 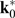, we obtain the results shown in Figure 7B.

Combining the information in Figures 7A and 7B, we obtain a good impression of the robustness region of the system. Contrary to the findings in [8], [10], [11], we conclude that the LL model has a large robustness region with a quite irregular shape.

## Discussion

An important question in the modeling of biological systems is for which parameter values the model has a stable limit cycle, since this is often the parameter range in which the model describes reality. In the literature [8], [10], [11], one mostly studies this topic by analyzing the eigenvalues of the Jacobian matrix of the equilibrium points of the model. For example, if some of these eigenvalues become purely imaginary, a so-called Hopf bifurcation takes place and a limit cycle comes into existence. However, analysis of eigenvalues of a Jacobian matrix is not the most appropriate way to study this problem, since these eigenvalues yield only local information. In the present paper we have presented a method to construct an estimate for the so-called robustness region in parameter space. The approach that we follow has a global, rather than a local character. Within a robustness region the system possesses a stable limit cycle and on its boundaries the system undergoes a bifurcation. A bifurcation is a dramatic change in the system dynamics indicating that the system is no longer robust if the parameters are perturbed further. For the present method, these bifurcations may be of any type and different parts of the boundary may be connected to different bifurcations.

The present method has especially been designed to scan robustness regions of systems with a high-dimensional parameter space. Its power stems from the fact that it scales linearly with the number of parameters. This implies that it is highly efficient from a numerical point of view. The present approach is based on observing the behavior of the Floquet multipliers of the periodic solution if the systems parameters are changed. In this way, one easily detects all bifurcations that may occur to the periodic solution, such as Hopf, fold, flip, and Neimark-Sacker bifurcations, which lead to disappearance or period doubling of the periodic solution.

The method has first been tested for low-dimensional systems. It is shown that for a 2-dimensional parameter space, the results are in full agreement with those obtained by the package AUTO. Thereafter the method has been applied to a high-dimensional system, the Laub-Loomis model which has 14 parameters. In this case, the method appears to be highly efficient, indeed. Contrary to the results reported in the literature [8], [10], [11], the method yields an estimate that is very big and irregularly shaped. The latter means that the Laub-Loomis model is much more robust with respect to changes in one parameter than in another. The present approach yields this information and is as such an extension of the methods available in literature. In the present method, a first direction is chosen such that the Floquet multipliers will change mostly if the continuation is applied along this direction. The approach finds axes that together span the estimated region.

Since all information about the limit cycle along the used axes becomes available, it requires no extra work to present, e.g., level line plots of the period of the limit cycle. Together with the general types of bifurcation that are detected, this provides a reliable and insightful impression of the dynamical behavior of a model in a wide range of values around a nominal point.

## Materials and Methods

### Floquet Theory and Periodic Solution

Consider an ordinary differential equation system
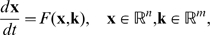
(7)where 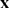 denotes the vector of state variables and 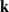 the vector of parameters. Suppose that this system has a stable periodic solution at 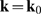 with periodic solution 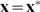 and period 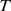.

In order to investigate the stability of the solution, we linearize around the periodic orbit 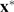 and obtain
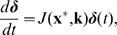
(8)where 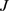 is the Jacobian matrix of (7) with respect to its state variables 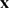. Since 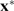 is 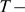
periodic, the Jacobian matrix 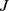 is also 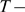
periodic. According to Floquet theory (see [13] and [14]), the fundamental solution of (8), which is a matrix that is composed of 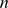 independent solutions of (8), can be written as
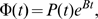
(9)with 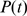 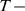
periodic and 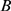 a constant 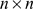 matrix. Thus,
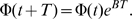
(10)


Here, 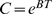 is called the monodromy matrix of the system and the eigenvalues of 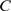 are called the Floquet multipliers of the system. One of them is always real and equal to 1. A necessary and sufficient condition for the periodic solution of (8) to be stable is that the other 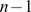 multipliers have modulus less than 1, i.e. they lie inside the unit circle. The calculation of 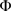 is explained underneath.

**Figure 10:**
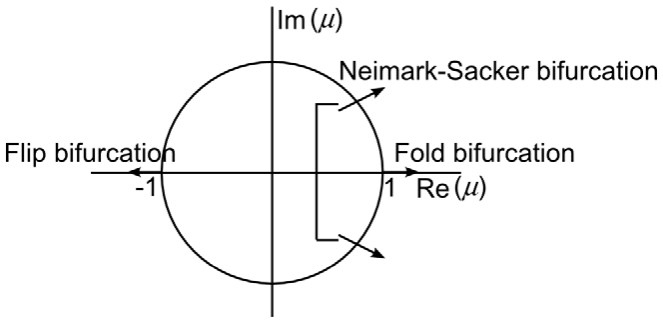
Limit cycle bifurcations according to the position of Floquet multipliers in the complex plane [19]–[21].

Three cases may be discerned [19]–[21], as illustrated in Figure 10:

1. A multiplier leaves the unit circle at 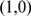. In this case, the model experiences a fold bifurcation.
2. A multiplier leaves the unit circle at 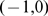. In this case, a flip bifurcation takes place and period doubling occurs.
3. Two conjugate multipliers cross the unit circle. In this case, a Neimark-Sacker bifurcation occurs.

### Calculation of Periodic Solutions

There are many methods discussed in the literature to approximate a periodic solution. To mention some of them: finite difference method, shooting method, and Poincare map method [21]. In this paper, we use the finite difference method because of its simplicity, and a short outline of the method is given below.

Consider again the ODE system (7). With the scaling
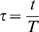
(11)with 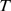 the period, the system reads as
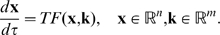
(12)


Now, (12) has to be solved in the time interval 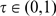. This time interval is discretized into 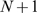 points with a uniform time step 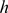:
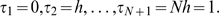
The solution of (12) at time steps 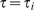 and 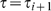 are related by
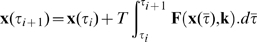
(13)Using the trapezoidal rule to represent the integral, we obtain
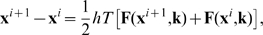
(14)where 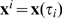. Since the system is periodic, 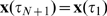, or
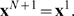
(15)Therefore, we have 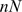 algebraic equations from (14) with 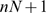 unknowns:
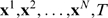



Finally, since the system that we consider is autonomous, the system is invariant to a linear shift in the time origin. To remove the arbitrariness of the phase, we specify the value of one component at 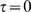, for example
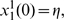
(16)where the value 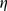 should be within the periodic solution of 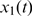. Thus, at time 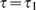 we have 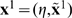 with 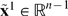. By imposing this condition, we have 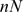 unknowns
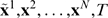
(17)and 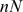 algebraic equations. Its solution can be found using, e.g., Newton's scheme, provided (16) is in the orbit of 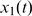. The details of this method can be found in [21].

So, we obtain the periodic solution in 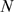 discretized points and the value of the period 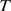 becomes known. The full periodic solution 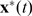 can then be obtained by integrating
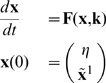
(18)numerically from time 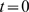 to 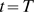.

#### Computing Floquet Multipliers

Let us consider the principal fundamental problem, i.e. problem (8) with now 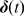 taken to be a matrix
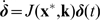
(19)with initial values
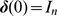
(20)where 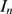 is the 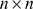 identity matrix. The Floquet multipliers of the system can then be obtained by integrating the above equation for one period, that is from 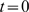 to 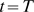. Then, the Floquet multipliers, denoted by 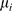, 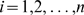, are the eigenvalues of the matrix 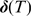.

Note that if we employ the same numerical technique to integrate (18) and (19), both systems can be solved simultaneously. We denote the 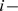
th column of the matrix 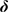 by 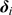 and solve
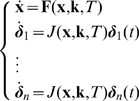
(21)with initial conditions
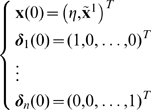
(22)


Since one of the multipliers should be real and equal to 1, the approximation of the periodic solution and the Floquet multipliers are carried out iteratively. If no multipliers are close to 1, we increase the number 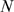 and solve again (14) and (21) until one of the multipliers is close to 1 within a prespecified accuracy.

### Continuation Method

We start at a nominal point 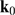 in parameter space, where the model has a stable limit cycle, so that the Floquet multipliers lie within the unit circle (except for one). The approach outlined here is also applicable if 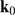 lies on the boundary of the robustness region. The first direction 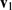, the construction of which is described below, will then point into the robustness region. It suffices to follow that direction until the boundary at the other side is met in a point 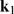, say, and to choose as new nominal point the midpoint of 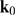 and 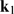. The next step is to perturb the nominal point 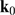 along 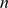 orthogonal directions 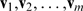.

To construct 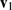, we introduce the function
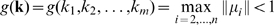
(23)which is nothing else but the largest modulus multiplier in 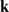 that is less than 1. The gradient
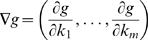
(24)is calculated numerically by
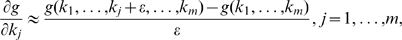
(25)taking 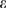 smaller and smaller until convergence is reached.

For the first direction 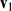, we now take 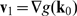. For the other perturbation directions we choose vectors that are orthogonal to 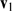 and to each other. They are calculated by the Gram-Schmidt method. The set of perturbation directions is thus
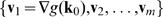
(26)Note that the choice of 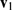 is unique, but the choice of 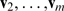 is not. However, the resulting approximate for the robustness region does not much depend on this choice, unless this region is highly irregularly shaped. To check the outcome it is recommendable to apply the method with a number of different nominal points and compare the outcomes. This will give a very good impression of the situation in parameter space.

The idea is now to perturb the nominal parameters 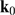 along these directions, so for direction 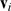, we walk along the line
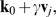
(27)with 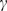 both positive and negative and check for which 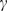 we approach a bifurcation. This yields the principal axes of the estimated robustness region.

An improvement of this concept is obtained by repeating this procedure but with 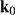 replaced by, e.g., the center of the longest axis. This leads to a refined approximation of the full robustness region. This idea is shown in Figure 1, where the initial nominal point 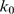 is shifted to 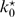 and the direction given by the line CE has been added. By another shift or by taking extra directions, this estimate can easily be improved.

### Algorithm

**Figure 11:**
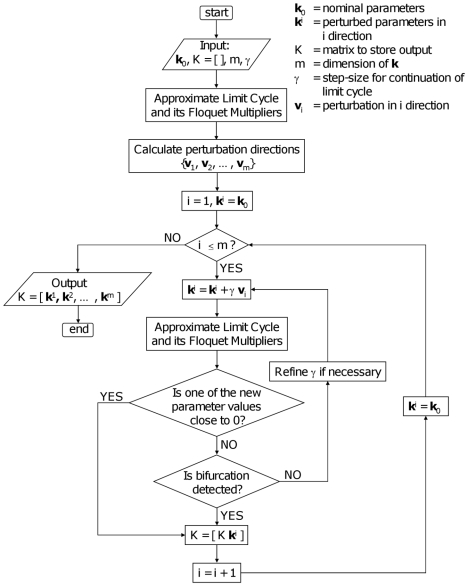
Flow chart of the method to approximate the robustness region around a nominal point 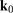. The approximated region is obtained by scanning the parameter space along orthogonal directions starting at 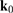.

In Figure 11 the flow chart of the algorithm is given. In this diagram we point out in a concise way that the algorithm contains the following steps:

1. Calculate the perturbation directions 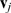 at the nominal parameter 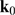. For 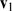, take 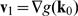 using (25) and construct the other perturbation directions using the Gram-Schmidt method.
2. Calculate the periodic solution and its multipliers along the lines (27) starting from 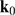. If one or more multipliers pass the unit circle, a bifurcation has been detected.
3. If refinement is required, move the nominal point to the center of the longest axis and repeat the procedure. Also, extra directions could be chosen.

## References

[1] Kaneko K (2006). Life: an introduction to complex systems biology.

[2] Alon U (2006). An introduction to systems biology: design principles of biological circuits.

[3] Kitano H (2004). Biological robustness.. Nature Reviews Genetics.

[4] Klipp E, Herwig R, Kowald A, Wierling C, Lehrach H (2005). Systems biology in practice: concepts, implementation and application.

[5] Morohashi M, Winn AE, Borisuk MT, Boluri H, Doyle J (2002). Robustness as a measure of plausibility in models of biochemical networks.. Journal of Theoretical Biology.

[6] Laub MT, Loomis WF (1998). A molecular network that produces spontaneous oscillations in excitable cells of *Dictyostelium*.. Molecular biology of the cell.

[7] Doedel E, Paffenroth R, Champneys A, Fairgrieve T, Kusnetsov Y (2007). Auto 07p: Continuation and bifurcation software for ordinary differential equations..

[8] Ma L, Iglesias PA (2002). Quantifying robustness of biochemical network models.. BMC Bioinformatics.

[9] Zhou K, Doyle J, Glover K (1996). Robust and optimal control.

[10] Kim J, Bates DG, Postlethwaite I, Ma L, Iglesias PA (2006). Robustness analysis of biochemical network models.. IEE Proceedings - Systems Biology.

[11] Ghaemi R, Sun J, Iglesias PA, Del Vecchio D (2009). A method for determining the robustness of bio-molecular oscillator models.. BMC Systems Biology.

[12] Barabási AL, Oltvai ZN (2004). Network biology: understanding the cell's functional organization.. Nature Reviews Genetics.

[13] Verhulst F (1996). Nonlinear differential equations and dynamical systems.

[14] Mattheij R, Molenaar J (2002). Ordinary differential equations in theory and practice.

[15] Rosenzweig ML, MacArthur RH (1963). Graphical representation and stability conditions of predator-prey interactions.. The American Naturalist.

[16] Klebanoff A, Hastings A (1994). Chaos in three species food chains.. Journal of Mathematical Biology.

[17] Kuznetsov YA, Rinaldi S (1996). Remarks on food chain dynamics.. Mathematical Biosciences.

[18] Boer MP, Kooi BW, Kooijman SALM (2001). Multiple attractors and boundary crises in a tri-trophic food chain.. Mathematical Biosciences.

[19] Kuznetsov YA (2004). Elements of applied bifurcation theory.

[20] Torrini G, Genesio R, Tesi A (1998). On the computation of characteristic multipliers for predicting limit cycle bifurcations.. Chaos, Solitons & Fractals.

[21] Nayfeh A, Balachandran B, Rand R (2004). Applied nonlinear dynamics: analytical, computational, and experimental methods.

